# The effect of popliteal sciatic nerve block with methylprednisolone in the management of postinjection sciatic neuropathy after clinical and electrophysiological examination

**DOI:** 10.3906/sag-2009-196

**Published:** 2021-10-21

**Authors:** Damla YÜRÜK, Ayçin YILDIZ TABAKOĞLU

**Affiliations:** 1 Division of Algology, Department of Neurology, University of Health Sciences Diskapi Yıldırım Beyazıt Research&Training Hospital , Ankara Turkey; 2 Division of Neurophysiology, Department of Neurology, University of Health Sciences Yüksek İhtisas Research&Training Hospital , Bursa Turkey

**Keywords:** Sciatic neuropathy, postinjection neuropathy, popliteal block

## Abstract

**Background/aim:**

This study aimed to investigate the effects of popliteal sciatic nerve block (PSNB) in the treatment of postinjection sciatic neuropathy (PISN) resistant to conservative treatments.

**Materials and methods:**

Patients diagnosed with PISN were included in the study. A damaged branch of the sciatic nerve was detected after neurological and electrophysiologic studies (EPSs). Visual analogue scale (VAS) was administered before, one hour after, and one month after the procedure. Also Leeds Assessment of Neuropathic Symptoms and Signs pain scale (LANSS) was administered before and one month after the procedure. The effects of EPSs findings and loss of muscle strength on the VAS and LANSS scores that measured after PSNB were evaluated.

**Results:**

PSNB was performed in 17 patients (12 males and 5 females) with a diagnosis of PISN. Their mean age was 54.95 ± 12.55 years, and the mean duration of symptoms was 3.53 ± 1.28 months. The EPS findings revealed a lateral truncus injury in 5, a medial truncus injury in 3, and injury to both in 9 patients. The initial muscle power scale scores were grade 0 in 2, grade 2 in 1, grade 3 in 7, grade 4 in 5, and grade 5 in 2 patients. The initial VAS and LANSS scores were 7.53 ± 1.06 and 17.35 ± 3.12. The mean VAS scores at the first hour and one month after the procedure decreased to 2.53 ± 1.70 and 4.18 ± 1.74 while the mean LANSS score one month after the procedure was reduced to 7.88 ± 5.84. The effects of EPSs findings and loss of muscle strength were found significant (p = 0.001), but the duration of symptoms was not found significant (p = 0.36) on the VAS and LANSS scores that measured after PSNB.

**Conclusion:**

The outcome of this research proved the effectiveness of PSNB with methylprednisolone in the management of PISN, especially in patients whose pain was located below the knee. EPSs findings and loss of muscle strength indicated the severity of the nerve damage affect the success of PSNB in pain management, but the length of time that elapsed after the nerve injury did not.

## 1. Introduction 

The most common injury mechanism that affects the sciatic nerve is receiving an intramuscular injection in the gluteal region, which is an iatrogenic cause with a frequency of 28% [1]. Direct trauma from the needle is often associated with the use of a more medial and/or inferior site for the injection [2]. There is a consensus the upper outer region of the buttock is the most reliable location for an injection [3]. In addition, the patient’s subcutaneous tissue and gluteus muscle thickness in this area are effective at preventing sciatic nerve injury after intramuscular injection [4]. The other mechanism for postinjection sciatic neuropathy (PISN) is nerve fiber damage induced by neurotoxic chemicals in the agent injected. The most common intramuscularly delivered agents that are injected into the nerve are analgesics, antiemetics, antibiotics, vitamins, vaccines, and steroid drugs [5].

The clinical presentation of a sciatic nerve injury includes immediate electric-like shock sensations down to the extremity. Concomitantly, patients often report the onset of variable motor and sensory deficits with pain from the injection site in the buttock that extends over the knee and may even be felt in the foot [6].

Electrophysiologic studies (EPSs) are more sensitive than a physical examination, which makes them invaluable in helping to define the location and to grade the severity of the lesion, exclude other lesions, and predict recovery [7]. EPSs are best performed three or more weeks after the injury since Wallerian degeneration will have been completed by this time.

Recommended treatments for PISN include surgical and nonsurgical methods including the administration of drugs and physical therapy. Physiotherapy is often required, but patients have difficulty completing early physiotherapy due to severe pain. Adequate pain management may not be possible in some patients when the time required for the drug to reach the effective dose is long or when the dose cannot be increased due to side effects. However, performing physiotherapy a long time after the nerve damage occurs may lead to permanent neurological deficits [8].

Successful reduction of pain in PISN increases compliance with physiotherapy in patients so that impaired motor and sensory functions can be restored. Therefore, the purpose of this study was to present popliteal sciatic nerve block (PSNB) therapy for pain management in PISN that is resistant to conservative treatments. 

## 2. Materials and methods

### 2.1 Participants

Twenty patients who complained of varying degrees of pain, loss of sensation, and weakness in the lower extremity following gluteal injection who visited the Algology Clinic of Health Sciences University at the Yüksek İhtisas Research and Training Hospital in Bursa between January and June 2019 were included in this study. The diagnosis of PISN was based on the patient’s clinical history, neurological examination, and EPSs findings.

The inclusion criteria were as follows: 1) pain that extends from the knee to the foot, which is certain to develop after gluteal injection, 2) pain that is resistant to drug therapy and a VAS score > 3, 3) damage detection in the medial or lateral truncus of the sciatic nerve on EPSs. The exclusion criteria were as follows: 1) the presence of lumbar radiculopathy and other causes of sciatica, 2) pain that extended from the gluteal region, 3) successful pain therapy with drugs and a VAS score ≤ 3; and 4) the use of anticoagulants. 

This prospective randomized controlled study received approval by the Local Ethics Committee (Health Sciences University of the Bursa Yüksek İhtisas Research and Training Hospital Clinical Research Ethics Committee, Decision Number 2011-KAEK-25 2018/09-7). After being given information about the study, written informed consent was obtained from all participants.

### 2.2. Study design

**Figure 1:**
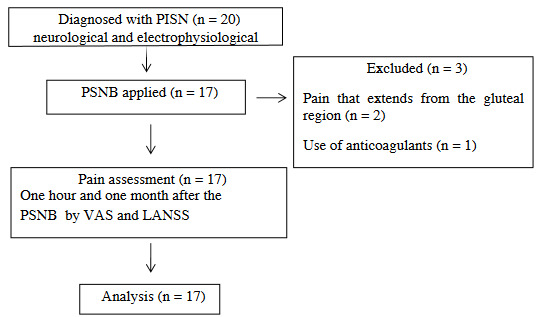
Design of the study. PISN, postinjection sciatic neuropathy; PSNB, popliteal sciatic nerve block; VAS, Visual Analog Scale; LANSS, Leeds Assessment of Neuropathic Symptoms and Signs.

A neurophysiology specialist physician performed the neurological examinations and EPSs in all patients and made the diagnosis of PISN. EPSs were done between three weeks and six months after the nerve injury. A pain physician identified the patients suitable for PSNB and performed the procedure using ultrasound guidance. The patients were allowed to continue their drug therapy at the same dose during the study. Demographic data, duration of symptoms, causes of pain, pain localization, neurological examination findings, and nerve damage detected by the EPS were recorded. Visual analogue scale (VAS) was administered before, one hour after, and one month after the procedure. Also Leeds Assessment of Neuropathic Symptoms and Signs pain scale (LANSS) was administered before and one month after the procedure. The study design is illustrated in Figure 1.

### 2.3. Intervention

Blocks were performed in an operating room by a pain physician with a 12–18 MHz linear ultrasound transducer (MyLab 30; Esaote SpA, Genoa, Italy) under sterile conditions with the patient placed in a prone position. Intravenous access, supplemental oxygen, and standard monitoring (electrocardiogram, noninvasive blood pressure monitoring, and pulse oximetry) were established in all patients. None of the patients experienced complications during or after the procedure.

The PSNB technique was as follows. A linear ultrasound transducer was placed in the transverse position at the popliteal crease, and then the popliteal artery and vein were identified. The biceps femoris muscle was observed lateral to the popliteal artery, and the semimembranosus and semitendinosus muscles were identified in the medial plane. The tibial nerve was seen as a hyperechoic, rounded structure lateral to the popliteal vein. The common peroneal nerve was visible lateral to the tibial nerve. The transducer was advanced proximally until the tibial and peroneal nerves that come together to form the sciatic nerve were visualized before dividing. A 10-cm, 22-G Stimuplex needle (B. Braun Medical Ltd., Melsungen, Germany) was inserted from 2–3 cm lateral to the transducer using an in-plane approach from a lateral to medial direct. The needle tip was contacted with either branch of the nerve; during nerve stimulation (0.5 mA for 0.1 ms), a motor response of the calf or foot was observed. The needle tip was then placed into the space between the two components of the sciatic nerve that were slightly separated by adipose tissue. It was observed that 80 mg methylprednisolone with 2% lidocaine in a 10-mL solution was distributed in the epineural sheath, and the tibial and peroneal nerves were separated.

The injection site was pressed to achieve hemostasis. Sensory and motor examinations were performed to ensure a successful block assessment. Sensory examinations of the dorsum of the foot for the common peroneal nerve and the middle part of the foot for the tibial nerve were performed. The plantar dorsiflexion strength of the ankle was evaluated during a motor examination.

### 2.4. Evaluation parameters

The severity of pain along the sciatic nerve distribution was evaluated using the VAS, which was developed by Price et al. [9]. Patients rated their pain on a scale of 0–10, with 0 representing no pain and 10 representing the worst pain imaginable. The pain assessment was performed before, one hour after, and one month after the PSNB by the VAS.

The amount of neuropathic pain was confirmed with the Turkish version of the LANSS pain scale [10]. This scale has a 24 total possible points; a LANSS score ≥12 indicates that neuropathic pain mechanisms are effective, while a LANSS score <12 signifies that current neuropathic pain mechanisms are not effective.

### 2.5. Statistical analysis

In this study, the descriptive statistics were expressed as follows: the mean ± standard deviation (SD) for continuous variables with a normal distribution; the median (interquartile range) for continuous variables without a normal distribution; frequencies and percentages for categorical variables. To determine the normality of continuous variables, the results of the Shapiro–Wilk test were evaluated. The change in VAS score over time was evaluated using the Friedman test. The difference between the initial and final LANSS score was examined with the Wilcoxon test. The significance level was set as p < 0.05. The data analysis was performed using the Statistical Package for the Social Sciences (SPSS) software, version 23.0 (IBM Corporation, Armonk, NY, US).

## 3. Results

PSNB was performed in 17 patients diagnosed with PISN: 12 (70.6%) males and 5 (29.4%) females. Their mean age was 54.95 ± 12.55 (range: 27–75) years, and the mean duration of symptoms was 3.53 ± 1.28 (2–6) months. (Table 1)

**Table 1:** Baseline characteristics of patients at the initial assessment.

Variable	Patients N = 17
Age (years)*	54.95 ± 12.55 (27–75)
SexMaleᵟ	n =12 (70.6%)
Femaleᵟ	n = 5 (29.4%)
Duration of symptomsᵟ≤3 months	n = 11 (64.7%)
>3 months	n = 6 (35.3%)
VAS baseline*	7.53 ± 1.06 (6–9)
LANSS baseline*	17.35 ± 3.12 (13–24)
EPSs findingsᵟLateral truncus	n = 5 (29.4%)
Medial truncus	n = 3 (17.6%)
Both trunci	n = 9 (52.9%)
Muscle power scaleᵟGrade 0	n = 2 (11.8%)
Grade 1	n = 0 (0%)
Grade 2	n = 1 (5.9%)
Grade 3	n = 7 (41.2%)
Grade 4	n = 5 (29.4%)
Grade 5	n = 2 (11.8%)
Injection contentᵟAnalgesic	n = 14 (82.4%)
Vitamin	n = 2 (11.8%)
Antibiotic	n = 1 (5.9%)

The values are presented * as mean +/- standard deviation (min-max) and ᵟ as n (%). VAS: Visual Analog Scale; LANSS: Leeds Assessment of Neuropathic Symptoms and Signs pain scale; EPSs: Electrophysiologic studies.

The EPS findings revealed lateral truncus injury in 5 (29.4%), medial truncus injury in 3 (17.6%), and both types of truncus injury in 9 (52.9%) patients. In 6 of these 9 (52.9%) patients, severe partial or full denervation in the muscles innervated from the lateral trunk was found, and mild partial denervation in the muscles innervated from the medial trunk was observed. In 3 of these 9 (52.9%) patients, severe partial denervation or full denervation in the muscles innervated by both trunci were recorded. Of the 5 patients with only a lateral trunk deficit, 2 had mild partial denervation, while 3 exhibited complete denervation of the muscles innervated by the lateral trunk. In 3 patients with only a medial trunk deficit, complete denervation was observed in the muscles innervated by the medial trunk. (Table 1)

Based on their clinical features, all patients had severe pain accompanied by sensory symptoms. The initial VAS and LANSS scores were 7.53 ± 1.06 (range: 6–9) and 17.35 ± 3.12 (range: 13–24). Except for 2 patients with only sensory symptoms, all patients had drop foot. The initial MRC Muscle Power Scale classifications were grade 0 in 2 (11.8%), grade 2 in 1 (5.9%), grade 3 in 7 (41.2%), grade 4 in 5 (29.4%), and grade 5 in 2 (11.8%) patients. The type of injection that caused the PISN was an analgesic in 14 (82.4%), a vitamin in 2 (11.8%), and an antibiotic in 1 (5.9%) patient. (Table 1)

**Figure 2:**
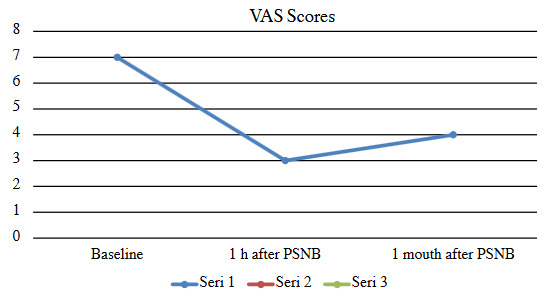
Trends in the change in VAS scores in patients with PISN who underwent PSNB. VAS: Visual Analog Scale; PISN: postinjection sciatic neuropathy, PSNB: popliteal sciatic nerve block

**Figure 3:**
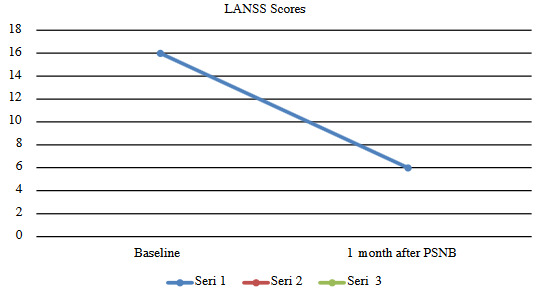
Trends in the change in LANSS scores in patients with PISN who underwent PSNB.

The median VAS scores at the first hour and one month after the PSNB were 3 and 4. The pairwise comparison of VAS scores measured after 1 h and before (p = 0.000), 1 month after and before (p = 0.008), after 1 h and after 1 month (p = 0.039) were found to be significant. The median LANSS score at before and one month after the PSNB was 16 and 6. LANSS differenced measured before and 1 month after PSNB (p = 0.000) was found to be significant (Table 2, Figure 2 and 3).

**Table 2:** The change and comprasion of VAS score over time.

	Median	Percentile 25	Percentile 75	Test Statistic	StandardError	p value
VAS scoreBefore PSNB	7	7	8			
1 Hour after PSNB	3	2	4			
1 Month after PSNB	4	3	5			
1 Hour after PSNB-Before PSNB				1.882	0.343	0.000*
1 Month after PSNB-Before PSNB				1.029	0.343	0.008*
1 Month hour PSNB-1 Month after PSNB				–0.853	0.343	0.039*
LANSS scoreBefore PSNB	16	15	19			
1 Month after PSNB	6	5	11			
1 Month after PSNB-Before PSNB						0.000**

*Pairwise comparison of VAS scores with Friedman test. *** Wilcoxon test.

LANSS: Leeds Assessment of Neuropathic Symptoms and Signs pain scale, PSNB: popliteal sciatic nerve block

The mean VAS scores before and one month after the PSNB in the patients with lateral or medial truncus injury were 6.75 ± 0.46 and 3.00 ± 0.75, while patients with both types of truncus injury were 8.22 ± 0.97 and 5.22 ± 1.71. The mean LANSS before and one month after the PSNB in the patients with lateral or medial truncus injury were 14.88 ± 1.24 and 19.56 ± 2.55, while patients with both types of truncus injury were 3.88 ± 2.53 and 11.44 ± 5.68. (Table 3) 

**Table 3:** VAS and LANSS scores before and 1 month after PSNB according to EPS findings, duration of symptoms, and loss of muscle strength.

	VASBefore	VAS1 month after	LANSSBefore	LANSS 1 month after
EPS findings Lateral or medial truncus	6.75 ± 0.46	3.00 ± 0.75	14.88 ± 1.24	3.88 ± 2.53
Both trunci	8.22 ± 0.97	5.22 ± 1.71	19.56 ± 2.55	11.44 ± 5.68
Duration of symptoms≤3months	7.64 ± 1.02	3.64 ± 1.12	17.36 ± 2.90	6.91 ± 4.23
>3months	7.33 ± 1.21	5.17 ± 2.31	17.33 ± 3.77	9.67 ± 8.21
Loss of muscle strengthMaximum (MRC 3,4,5)	8.20 ± 0.78	5.00 ± 1.764	19.10 ± 2.80	10.80 ± 5.73
Minimum (MRC 0,1,2)	6.57 ± 0.53	3.00 ± 0.816	14.86 ± 1.34	3.71 ± 2.69

The values are presented as mean+/-SD (min-max).

The mean VAS scores before and one month after the PSNB in the patients with duration of symptoms ≤3 months were 7.64 ± 1.02 and 3.64 ± 1.12, while patients with a duration of symptoms >3 months were 7.33 ± 1.21 and 5.17 ± 2.31. The mean LANSS before and one month after the PSNB in the patients with a duration of symptoms ≤3 months were 17.36 ± 2.90 and 6.91 ± 4.23, while patients with a duration of symptoms >3 months were 17.33 ± 3.77 and 9.67 ± 8.21. (Table 3)

The mean VAS scores before and one month after the PSNB in the patients with MRC of 3,4,5 (maximum) were 8.20 ± 0.78 and 5.00 ± 1.764, while patients with MRC score of 0,1,2 (minimum) were 6.57 ± 0.53 and 3.00 ± 0.816, respectively .The mean LANSS before and one month after the PSNB in the patients with MRC of 3,4,5 (maximum) were 19.10 ± 2.80 and 10.80 ± 5.73 , while patients with MRC of 0,1,2 (minimum) were 14.86 ± 1.34 and 3.71 ± 2.69. (Table 3)

The effects of EPSs findings (p=0.001) and loss of muscle strength (p = 0.001) were found significance, but the duration of symptoms (p=0.36) was not found significant on the VAS scores that measured after PSNB. And also the effects of EPSs findings (p = 0.001) and loss of muscle strength (p=0.004) were found significance, but the duration of symptoms (p = 0.55) was not found significant on the VAS scores that measured after PSNB (Table 4)

**Table 4:** The effect of EPS findings, duration of symptoms, and loss of muscle strength

	Mean Square	F test	p value
VAS 1 Month after PSNB –Before PSNBEPS findings	28.904	18.170	0.001*
Duration of symptoms	2.924	0.880	0.363*
Loss of muscle strength	27.108	15.848	0.001*
LANSS 1 Month after PSNB –Before PSNBEPS findings	317.779	16.009	0.001*
Duration of symptoms	14,439	0.360	0.557*
Loss of muscle strength	264.222	11.282	0.004*

*Tests of between-subjects effects.

## 4. Discussion

Many morphological and metabolic changes occur after trauma to the peripheral nerve. These changes take place not only in the region of damage but also in the nerve trunk, in segments proximal and distal to the location of the injury, and in the neuromuscular junction or sensory receptors where the nerve fiber ends [11]. The biggest difference between peripheral nerve injuries and other tissue injuries is demonstrated by Wallerian degeneration, which proceeds towards the neuronal structures distal to the lesion [12]. In patients with PISN, the pain initially extends from the hip to the foot and then may migrate from the knee to the foot or from the ankle to the foot, this phenomenon is likely due to Wallerian degeneration. 

Inflammatory reactions and mediators play an important role in nerve repair, but prolonged inflammation may negatively affect recovery and lead to the development of neuropathic pain [13]. The ectopic discharge of activity from the injured site up-regulates and sensitizes the nociceptors and thereby contributes to the development of central sensitization [14]. Corticosteroids may be an effective therapy for neuropathic pain because they inhibit pro-inflammatory cytokines, prostaglandin synthesis, neural firing, input to central neurons, and also neurogenic extravasation and perineural edema formation by reducing substance P at the site of the nerve injury [15,16]. Perineural corticosteroid injections also produce analgesia in a variety of pain-related disorders, including neuromas [17] and nerve entrapments [18]. Nevertheless, only a few trials have investigated perineural corticosteroid injections for PISN.

Trans-sacral block with corticosteroids through the unilateral S1-S2-S3 sacral foramina has been reported to induce recovery from pain with PISN [19,20]. In these case series, a corticosteroid nerve blockade was performed proximal to the damaged nerve, whereas, in our study, it was performed distal to the damaged nerve. We selected the location of the nerve block based on the results of neurological examinations and EPSs. We performed PSNB because pain localization did not cover the entire sciatic nerve dermatome but was mostly located in the tibial or peroneal nerve dermatome, which was confirmed with EPSs. In a study that investigated transcutaneous electrical nerve stimulation (TENS) for the treatment of PISN, placing the electrodes in the painful area was reported to be sufficient for maximum pain relief [21]. Similarly, in the present study, we performed PSNB because patients with PISN reported that their pain was localized below the knee. 

A previous study reported the outcomes of distal decompression of the peroneal nerve at the fibular tunnel following sciatic nerve injury secondary to total hip arthroplasty [22]. After performing peroneal nerve decompression at the fibular tunnel, 65% of the patients recovered dorsiflexion strength. The authors explained this finding as follows: Disruption of axoplasmic flow as a result of the nerve injury may also enlarge the nerve, further exacerbating potential compression at known sites of entrapment. Decompression of the peroneal nerve at the fibular tunnel may then also allow improvement when performing nerve glide exercises and also lessen existing tension, which may promote potential recovery.

The loss of motor and sensory function in the expected dermatome indicates the success of the PSNB, which is typically administered with 2% lidocaine. Complete pain relief one hour after the popliteal block depends on the amount of membrane stabilizing and the analgesic effects of the local anesthetic. After this local anesthetic wears off, the VAS and LANSS pain scores had increased one month after PSNB compared to only one hour after the PSNB. Even so, the VAS and LANSS pain scores one month later had decreased compared to the scores recorded before the PSNB due to the anti-inflammatory effect of the corticosteroid. We think that stronger and longer-term analgesia can be achieved with repetitive blocks or pulsed radiofrequency [23].

EPSs findings can vary from mild to severe involvement depending on the severity of the PISN [24]. In studies involving large case groups, the lateral trunk is often more severely affected because it is located more lateral to the sciatic nerve, where protective epineural connective tissue and blood vessels are more tense [25]. In our study, the lateral trunk was isolated or affected along with the medial trunk, which confirmed this hypothesis. 

Proximal nerve injuries above the knee are difficult to treat because before irreversible changes occur, sprouts must travel the long distance to reinnervate distal muscles. While recovery is expected with conservative treatment in partial function losses, if there is a complete or severe loss of function in one or both trunks of the sciatic nerve, spontaneous recovery does not occur, and surgical intervention is required [26]. In our study, patients with a greater severity of nerve damage on EPSs findings (injury to both trunci) and a loss of muscle strength (MRC 3,4,5) experienced a lower success of PSNB in pain management. 

## 5. Conclusion 

Although further studies involving a greater number of patients are necessary, our study indicated the effectiveness of PSNB with methylprednisolone in the management of PISN, especially in patients with pain that is below the knee. The effect of the proximity of the injection to the site of the nerve injury remains unknown, and further studies are needed. The length of time that elapsed after the nerve injury did not affect but EPSs findings and loss of muscle strength indicated the severity of the nerve damage affect the success of PSNB in pain management. PSNB can be applied before surgery in PISN patients who have not recovered with spontaneous or conservative treatment.

## Informed consent

The study protocol received institutional review board approval, and all participants gave informed consent in the format required by the relevant authorities and/or boards.
